# Historical surveys reveal a long‐term decline in muskrat populations

**DOI:** 10.1002/ece3.7588

**Published:** 2021-05-02

**Authors:** Carrie Sadowski, Jeff Bowman

**Affiliations:** ^1^ Wildlife Research and Monitoring Section Ontario Ministry of Natural Resources and Forestry Peterborough Canada

**Keywords:** fur harvest, muskrat, *Ondatra*, population decline, *Typha*, wetlands

## Abstract

The muskrat (*Ondatra zibethicus*) is an iconic species in Canada, valued for both its fur and its integral role in wetland ecosystems, and widely regarded for its perseverance. However, the resilience of this semiaquatic mammal seems to be in question now as increasing evidence points to widespread population declines. Recent analyses of harvest data across North America suggest a reduction in their numbers, but this has not been widely corroborated by population surveys. In this study we replicated historic muskrat house count surveys at two large Great Lakes coastal wetlands and present confirmation that declines in muskrat harvest correspond to actual declines in muskrat abundance. At the Point Pelee National Park marsh and the Matchedash Bay‐Gray Marsh wetland we found that mean muskrat house counts declined by 93% and 91% respectively between historic surveys 40–50 yrs ago and contemporary surveys over the past 7 yrs. The factors responsible for these dramatic declines remain unclear but there may be a relationship with changes in the habitat quality of these wetlands that have occurred over the same time frame. Not only is the loss of muskrats an issue for the resulting loss of the wetland ecosystem services they provide, but it may be an indication of broader marsh ecosystem degradation. As such, a scarcity of muskrats should be considered a red flag for the state of biodiversity in our wetlands. Continued surveys and ongoing research are needed to shed more light on the current status of muskrat populations and their marsh habitats across their native range.

## Graphical abstract

The muskrat (*Ondatra zibethicus*) plays a valuable role in wetland ecology and is a staple among fur harvesters in North America. We investigated trends in muskrat populations at two large Great Lakes coastal wetlands and found that muskrat abundance declined significantly over the past 40–50 yrs, providing confirmation that reported declines in muskrat harvest correspond to actual declines in muskrat abundance. Muskrat decline can result in a loss of ecosystem services and may also be an indication of broader wetland ecosystem degradation.

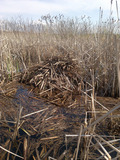

## 1 INTRODUCTION

The muskrat (*Ondatra zibethicus*) is seen as a fixture of wetlands across North America, being one of the most common and widely distributed furbearer species on the continent. They are found primarily in marshes, but also in ponds, sloughs, lakes, ditches, rivers, and streams from the east coast to the west and from the Mackenzie Delta in Canada's north to northern Mexico in the south (Boutin & Birkenholz, 1987). The muskrat also ranks as the top harvested wild furbearer in North America of the 20th century (Obbard et al., 1987) and has contributed more than any other animal to the combined income of North America's fur trappers over the past 150 yrs (OFMF, 2019). In the early 1900s, millions of muskrats were trapped and sold across North America, and though harvest numbers are lower today, muskrats remain a major source of income for fur trappers and are still among the most prevalent species trapped for fur (Fur Institute of Canada, 2019).

While the muskrat played a major role in the early fur trade and colonization of North America by Europeans (White et al., 2015), the species has been of cultural significance as a traditional clothing and food item and as a spiritual symbol among Indigenous people long before European explorers arrived on the continent. For example, in an Anishinaabe story of creation, the muskrat (Wa‐zhushk) comes to the rescue to help rebuild the Earth after a great flood and decimation of life, and is said to embody humility, courage, and determination (MacGregor, 2013).

As a wetland obligate and a significant consumer of marsh vegetation, the muskrat plays several important roles in wetland ecosystems. Their foraging, travel, and house‐building activities create numerous small openings in marshes, thereby increasing the interspersion of open water and emergent vegetation, which often results in increased structural diversity and plant species richness in wetlands (Connors et al., 2000; Keddy, 2010; Nyman et al., 1993). Different biotic communities are known to respond positively to such enhancements in habitat diversity (Wilcox & Meeker, 1992); in particular, a greater density and diversity of marsh birds and waterfowl have been found in wetlands with an equal ratio of open water to emergent vegetation (Kaminski & Prince, 1981; McDonnell, 1983; Weller & Fredrickson, 1973). Muskrat houses (both new and old) can also create important loafing and nesting sites for marsh birds. For example, the black tern (*Chlidonias niger*) will use muskrat houses and feeding platforms as nesting substrate (Hickey & Malecki, 1997) and it is quite common to find Canada geese (*Branta canadensis*) and sometimes trumpeter swans (*Cygnus buccinator*) loafing or nesting on old muskrat structures. Less recognized are the benefits that muskrats provide to snakes and turtles, many of which are species at risk. In an Illinois, USA, study, a large number of spotted turtle (*Clemmys guttata*) captures occurred in deep open water pools associated with muskrat lodges where muskrat grazing decreased the vegetative cover, and it was believed that these pools served as refugia for the turtles during periods of high temperature and/or drought (Litzgus & Brooks, 2000). Numerous vertebrate species, including more than ten herptile species, have been observed using muskrat houses, burrows, cleared pathways, and other features for thermoregulation, nesting, cover, and ease of travel (S. Gillingwater, unpublished data; Kiviat, 1978). Furthermore, muskrat activity can influence mussel abundance (Diggins & Stewart, 2000), invertebrate communities (De Szalay & Cassidy, 2001; Nummi et al., 2006), microbial activity (Wainscott et al., 1990), and nutrient cycling (Connors et al., 2000). Muskrats are also an important food item for many predators, such as red foxes (*Vulpes vulpes*), coyotes (*Canis latrans*), raccoons (*Procyon lotor*), raptors, and especially mink (*Neovison vison*) (McDonnell, 1983).

Early research on muskrats illustrated the density‐dependent nature of muskrat populations and a variety of abundance cycles (Clark & Kroeker, 1993; Erb et al., 2000; Errington, 1951). In their analysis of almost one hundred individual time series of muskrat harvest data from the Hudson's Bay Company in Canada, Erb et al. (2000) found that the mean period length of muskrat population cycles differed between ecozones, ranging from 3.7 to 8.6 yrs, with the shorter periods tending to occur at higher latitudes and in eastern regions. In some cases, however, the time series did not exhibit any periodicity. Evidence exists for both population‐intrinsic factors (e.g., social factors) and extrinsic factors (e.g., disease, environmental variability, and trophic interactions) explaining the observed patterns of these cycles (Errington, 1963 and Errington et al., 1963; Bulmer, 1974; Weller & Fredrickson, 1973). Despite some commonalities, these patterns lack consistency across geographic areas, and in many cases, muskrat population dynamics and their mechanisms of regulation remain unclear.

The muskrat is a prolific species, typically having 2–3 litters per year and an average litter size of 6.5 kits (Boutin & Birkenholz, 1987). As well, most muskrats are able to breed the same year they are born and have high dispersal capabilities. These demographic characteristics make muskrats relatively resilient to harvest and other population pressures (e.g., disease, predation) as a small number of individuals can quickly multiply and enable population recovery. They are also reasonably flexible in their habitat requirements, and as noted by Errington (1951), muskrats often demonstrate a remarkable ability to survive up to the very edges of what may be considered habitable range.

Today, however, this marsh denizen may not be thriving like it once was. There is a growing body of literature suggesting declines in muskrat populations from myriad locations across North America over the past 10–20 yrs. Most recently, Gregory et al. (2019) reported that muskrat harvest on Prince Edward Island declined by more than half when comparing the average harvest from 1977–1988 to 1988–2016. More broadly, Ahlers and Heske (2017) analyzed harvest data from 1970 to 2012 across the United States and, after controlling for pelt prices, found strong evidence that muskrat populations declined during this time period. Prior to that study, analyses by Roberts and Crimmins (2010) revealed a 75% decline in muskrat harvest across the northeastern United States and eastern Canada from 1986 to 2006. This decline was thought to be indicative of regional declines in muskrat abundance as the study's authors found that a previously strong correlation between harvest and pelt prices had weakened in the latter years of their analysis. In other words, they considered it was likely that recent changes in muskrat harvests were reflecting underlying population change (and not simply changes in harvest effort) because they did not find a strong relationship between harvest levels and pelt prices, which had previously defined the harvest dynamics of muskrats and other furbearer populations (Bailey, 1981; Scognamillo & Chamberlain, 2006). As well, in the same study the authors reported a lack of periodic fluctuations in the modern muskrat harvest data (as compared to the historic data, which exhibited mild periodicity), providing further support for widespread population decline (Roberts & Crimmins, 2010).

**FIGURE 1:**
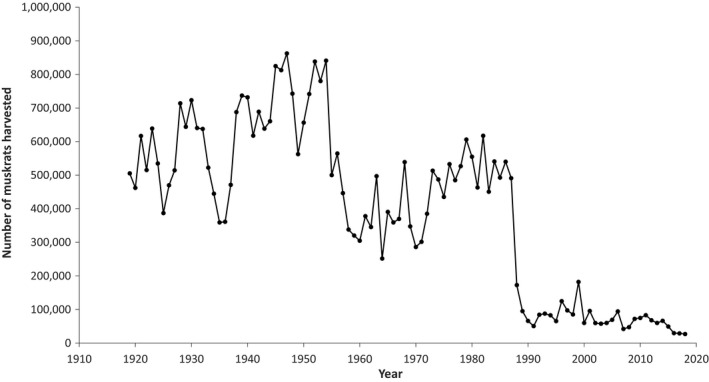
Total harvest of muskrats (*Ondatra zibethicus*) by trappers in Ontario from 1919 to 2018 (Statistics Canada Catalogue 23–013‐X and Ontario Ministry of Natural Resources and Forestry)

In Ontario, similar to trends across Canada, provincial fur harvest records show a clear decline in muskrat harvest over the past 100 yrs, most notably over the past 30 yrs (Figure 1). In fact, the average annual number of muskrats harvested in the past 30 yrs has declined by more than 90% from the mean in the previous 20‐yr period (late 1960s to late 1980s). While some of this decline can be explained by changes in fur harvest reporting procedures that occurred in Ontario in the late 1980s, as well as economic factors and cultural shifts in trapping, the magnitude and time span of the decline seem too great to be explained by these factors alone. For example, the spatial pattern of change in muskrat harvests from 1972 to 2004 suggests an actual decline in muskrat populations may have occurred because the pattern did not conform to the expectation arising from spatially homogeneous decline in fur price and therefore trapper effort (Gorman, 2007). Furthermore, the time span of the recent period of low muskrat harvest numbers (30 yrs) far exceeds that of the maximum population cycle length reported for muskrats (Erb et al., 2000). As well, the low annual numbers harvested in recent years are well below the lower limit of published historic muskrat population fluctuations (Statistics Canada, 2011).

Since many furbearers (including muskrats) are difficult and costly to census (Erb & Perry, 2003), harvest data are often the only type of information related to species’ population trends that are available for a long time series or across a large geographic area (White et al., 2015). However, there are problems with relying on harvest data to infer wildlife population trends, and these have been noted by many of the same authors that have analyzed harvest trends of furbearers (e.g., Gregory et al., 2019; Roberts & Crimmins, 2010). For example, harvest data can encompass trapping seasons of different lengths, can be missing data for some years, or can be deficient due to lack of full reporting. More importantly, harvest data can be biased by trapping effort, which can be affected by the number of trappers or by changing economic factors (e.g., pelt price) that might influence trapper behavior (Landholt & Genoways, 2000).

Although long‐term census data on muskrat populations are generally rare, a few researchers have published evidence of declines in local muskrat populations from direct count surveys. For example, Benoit and Askins (1999) reported that counts of muskrat houses from marshes of the Quinnipiac and Connecticut Rivers decreased dramatically (78% and 100%, respectively) between 1965 and 1990. As well, Ward and Gorelick (2018) analyzed muskrat house count records from 1970 to 2016 for the Peace‐Athabasca Delta in Canada and found a significant decline in population density over this time frame.

Other recent studies, while not long term in nature, have reported finding lower than expected muskrat densities in areas of seemingly suitable habitat. For example, Toner et al. (2010) reported a mean muskrat density of 0.71 houses/ha in six coastal wetlands along the Upper St. Lawrence River surveyed multiple times from 2001 to 2006, and Greenhorn et al. (2017) found a mean muskrat density of 0.27 houses/ha in 43 cattail‐dominated marshes surveyed along the north shore of Lake Ontario in 2014. In 2009, Gregory et al. (2019) intensively searched four marshes on Prince Edward Island for muskrats and reported a mean density of just 0.07 houses/ha. All of these results are much lower than the typical densities of 2.1–3.6 muskrat houses/ha reported previously in the literature for cattail‐dominated marshes in Canada (Messier & Virgil, 1992; Proulx & Gilbert, 1984).

Anecdotal and interview‐based reports of muskrat decline over the past few decades have also been made by trappers (Gregory et al., 2019), fur managers (Roberts & Crimmins, 2010), and Indigenous people across Canada (Brietzke, 2015; Straka et al., 2018; S. Mallany, personal communication, December 2016) and have also been received from a variety of sources by us. The underlying theme among these reports is that trappers and other long‐term land users are simply not finding muskrats in the numbers they used to, despite efforts to do so.

Collectively, these recent studies and reports point to potential widespread declines in muskrat populations. However, robust empirical data on long‐term trends in muskrat populations based on field observations are generally scarce in the literature. Thus, more information is needed to confirm that declines in muskrat harvest correspond to real declines in muskrat abundance (Ahlers & Heske, 2017). Fortunately, we learned of annual muskrat field surveys having been conducted at multiple locations in Ontario, Canada, between 1950 and 1990 and sought to exploit this untapped source of historic muskrat population data.

Our specific objectives in this study were to locate and revisit sites where historic muskrat survey data exist for Ontario, to replicate the historic survey methods as closely as possible, and to then compare contemporary survey results with the historic data to determine whether there have been empirical changes in the muskrat populations in these areas. We hypothesized that declines observed in muskrat harvest are due at least in part to real declines in muskrat populations. Therefore, we expected to observe evidence of fewer muskrats in contemporary versus historic muskrat surveys.

## 2 METHODS

### 2.1 Study area

**FIGURE 2:**
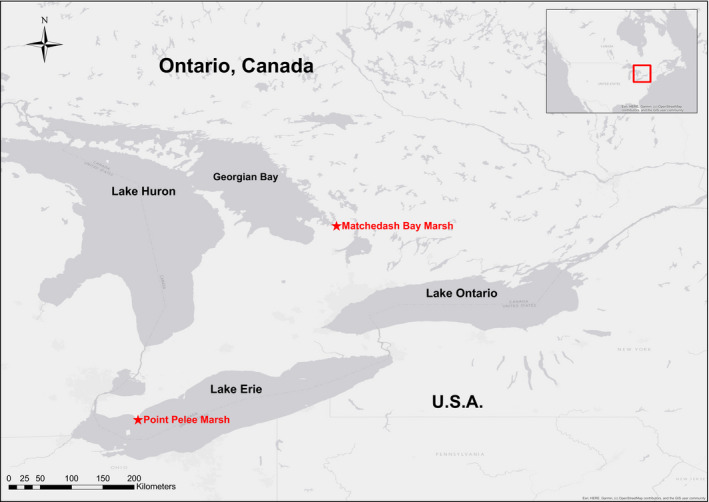
Study sites for historic and contemporary muskrat surveys in Ontario, Canada

We found two sites in Ontario with collections of multi‐year historic muskrat survey data from coastal wetlands: Point Pelee National Park and Matchedash Bay–Gray Marsh (Figure 2). In both cases, the historic data were more than 30 yrs old. The methods and data from these surveys are published in internal government reports, and we were able to obtain copies from the respective offices responsible for the resource management of these sites. We replicated the historic muskrat surveys in the marshes of these two locations between 2014 and 2019. Both sites are considered Great Lakes coastal wetlands but are of different hydrogeomorphic types; the Point Pelee Marsh is classified as a barrier‐beach wetland, while the Matchedash Bay complex is classified as a combination of protected embayment and drowned river mouth wetlands (Albert et al., 2005). These two sites are situated on different Great Lakes and are separated geographically by a straight‐line distance of 385 km.

#### 2.1.1 Point Pelee Marsh

The Point Pelee Marsh (41°57' N, 82°31' W; hereafter Pelee) occurs within a sandspit peninsula located on the north side of Lake Erie at the southernmost tip of Canada's mainland in the Carolinian forest zone (Lake Erie–Lake Ontario Ecoregion) of the Mixedwood Plains Ecozone (Crins et al., 2009). The marsh is approximately 1,100 ha in surface area (including large open water areas) and is designated as both a Provincially Significant Wetland in Ontario (MNRF, 2015) and a RAMSAR Wetland of International Significance by UNESCO. It is a protected area situated in Canada's smallest national park, which experiences over 200,000 visitors each year (Parks Canada, 2010). Muskrat trapping was historically a popular activity in the Pelee marsh but has not occurred there since 1958 when Parks Canada prohibited all trapping within the National Park (Menefy, 1969).

#### 2.1.2 Matchedash Bay–Gray Marsh

The Matchedash Bay and Gray Marsh wetland complex (44°45' N, 79°40' W; hereafter Matchedash) is located in central Ontario near the southeastern end of Lake Huron's Georgian Bay, spanning the boundary of the Georgian Bay and Lake Simcoe–Rideau Ecoregions at the interface of the Mixedwood Plains and Ontario Shield Ecozones (Crins et al., 2009). Similar to the Pelee wetland, the Matchedash wetland is approximately 1,100 ha in surface area (including large open water areas) and is designated as both a Provincially Significant Wetland in Ontario (MNRF, 2015) and a RAMSAR Wetland of International Significance by UNESCO (Wilson & Cheskey, 2001). Matchedash is primarily Crown Land (much of it designated and protected as a Provincial Wildlife Area), but there are several parcels owned and managed by Ducks Unlimited Canada under the Eastern Habitat Joint Venture Project (Ducks Unlimited, 2011). Licensed trapping is permitted and occurs throughout the wetland.

Both wetlands consist of approximately 700 ha of robust emergent marsh vegetation (MNRF, 2015), which is considered to be suitable muskrat habitat (Bellrose & Brown, 1941; Clark, 1994; Proulx & Gilbert, 1983). Cattails (*Typha* spp.) are by far the dominant plant species at both sites, comprising 89% of the marsh vegetation at Pelee (Markle et al., 2018) and representing the most common wetland cover type at Matchedash (Gartner Lee Limited, 1990). The invasive European common reed (*Phragmites australis ssp. australis*) is also present in each marsh (~ 6.5% of the marsh vegetation at Pelee; unknown coverage at Matchedash), along with relatively low coverage by narrow‐leaved emergents (e.g., sedges, grasses, and rushes), forb marsh, and graminoid meadow marsh community types (Gartner Lee Limited, 1990; Markle et al., 2018; Sadowski pers. obs.).

### 2.2 Survey methods

We aimed to replicate the historic survey methods as closely as possible to obtain comparable contemporary survey results. We undertook this task by following the detailed descriptions and survey maps provided in the old reports to relocate the same survey areas for each site. At Point Pelee National Park, muskrat surveys were initiated in the 1950s by Parks Canada staff and standardized in 1963 when the marsh was divided into 14 survey zones, with a 15th zone added in 1971 (Reive, 1978). The zones remained consistent in subsequent years; however, a few boundary adjustments were made in 1979 to accommodate changes in the vegetation structure of the marsh (Reive, 1979). The surveys were conducted annually until 1980, after which time no further muskrat surveys were undertaken (Bremner & Reive, 1980). At Matchedash Bay–Gray Marsh, muskrat surveys were initiated in late fall 1978 by Ontario Ministry of Natural Resources Midhurst (formerly Huronia) District staff and continued annually until 1986, with the exception of 1982 when no surveys were conducted (LaFrance, 1986).

**FIGURE 3:**
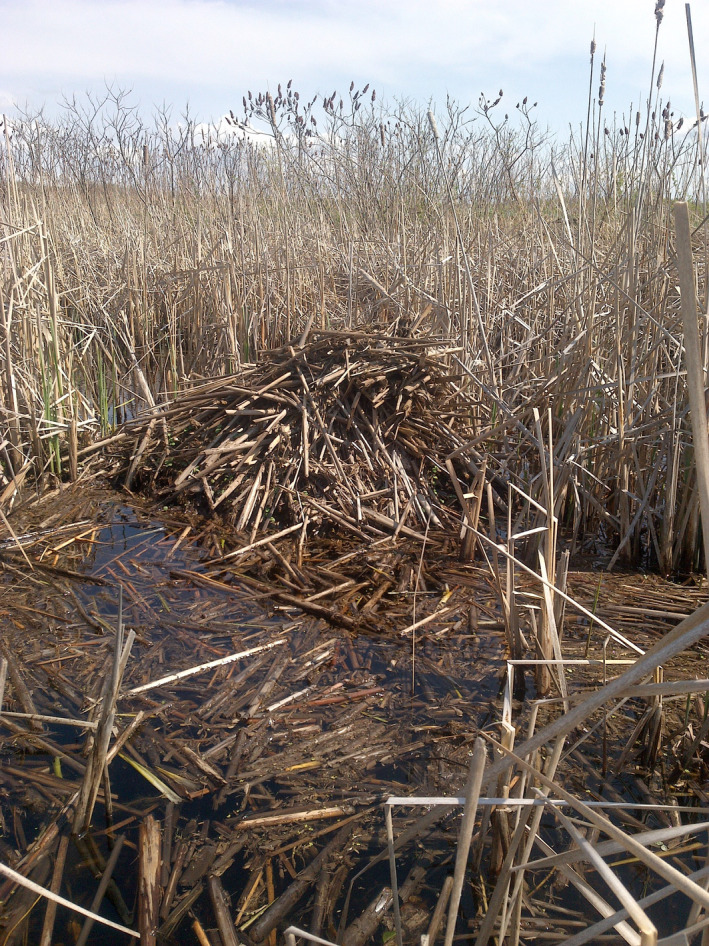
Muskrat house found during our survey of the Matchedash Bay–Gray Marsh in May 2018

At each site, the basic survey method was a count of all active muskrat houses (e.g., Figure 3) found within the respective search areas (survey zones) of the wetland. At Pelee, the survey method covered only a portion of the entire marsh (the perimeter of all ponds along the open water‐vegetation edge, which is typically where muskrat house building occurs), while at Matchedash the survey method aimed for a complete census of the entire marsh. We surveyed Pelee for muskrat houses during the winter of 2014 and 2015 and during the spring of 2017 and 2019 (four surveys spanning 6 yrs). We surveyed Matchedash for muskrat houses during the winter of 2014 and the springs of 2014–2018 (five consecutive survey years).

At Pelee, we conducted surveys the first 2 yrs in winter (March 2014 and February 2015) on foot with two observers walking on the ice along the inner edge of all marsh areas (i.e., the perimeter of mapped ponds, plus small channels and accessible pools) within each mapped survey zone, as depicted in the historic survey reports. Observers searched from the edge of the emergent vegetation to approximately 15 m inwards from the open water edge (or as far into the vegetation stands as we could detect muskrat houses) and counted all muskrat houses seen. This is the same survey method used in the historic surveys at Pelee, except that in some years the observers rode an ATV rather than walked. The latter 2 yrs of our surveys (2017 and 2019) were conducted by canoe in spring (with the exception of survey zone 15, which was conducted on foot in chest waders), as ice conditions were not adequate in the winter of those years to allow surveyors to safely traverse the marsh. The canoe‐based surveys followed the same routes covered on foot in winter the first two survey years; however, in each year a few survey zones were not covered due to access challenges. When surveys were conducted by canoe, the lead observer was stationed in the bow in a kneeling position and frequently stood up to ensure a comparable search height to the surveys conducted on foot in winter. As well, spring surveys were conducted in early spring before new growth and green‐up of the marsh vegetation so that visibility of muskrat houses was comparable to surveys conducted in winter.

At Matchedash, we conducted the very first survey in late winter (March 2014) on a combination of foot and snowmobile with two observers covering the entire marsh (both perimeter and interior areas); however, the snow cover was very deep (over 1.5 m) and we were concerned that muskrat houses might be buried and thus not detected. We verified this suspicion by returning to the site early the following spring (2 months later) and paddled the perimeter of the entire marsh by canoe, counting several muskrat houses that we missed due to snow depth in the winter. We know that these were not houses that were newly built in the interval between our winter and spring visits because we conducted the survey in May only a few weeks after ice‐out, which is not a time when muskrats conduct house building in this region. Muskrats typically build houses in the fall before freeze‐up, and these remain in place over the winter until they start to deteriorate (if no longer in use) the following spring–summer (Dozier, 1948). Over the following four years (2015–2018), we conducted all surveys at this site in May by canoe, as the Matchedash area typically receives heavy snow accumulation and thus we knew that detection of houses would continue to be hindered by snow cover in winter. The historic surveys, however, were conducted every year in winter by snowmobile (except for 1981 when it was conducted by boat in spring) and covered the entire marsh area, not just the perimeter of the vegetation stands, which the canoe‐based surveys were restricted to (even though this is where most muskrat houses typically occur). We compensated for this difference in survey coverage by using high‐resolution aerial photographs of the study area (collected ourselves in March 2018 during low snow cover conditions) to count muskrat houses in the areas we could not access by canoe. We then added our aerial count of houses found in these inaccessible areas of the marsh to our ground‐based counts from the rest of the marsh to produce a total site count for 2018 that we felt would be most comparable to the historic surveys with respect to marsh coverage. Next, we assumed that a similar proportion of inaccessible houses as determined from the 2018 imagery (compared with the ground‐based counts of accessible houses from the rest of the marsh) occurred in previous survey years, so we added this proportion to the previous four survey years’ house counts. This addition yielded a total site count for each year that should represent the most accurate final house count possible based on all available data. In other words, for each survey year we obtained a minimum count of muskrat houses observed from only our ground‐based survey, and an estimated maximum count based on a combination of the ground survey data and an adjustment derived from the 2018 imagery‐based survey. We considered the estimated maximum count as our best estimate for each year.

At Pelee, we also used imagery‐based house counts from 2017 and 2019 to supplement our ground counts in the survey zones we could not access or fully cover, similar to how we used the Matchedash imagery data. For this, we used high‐resolution imagery available from the County of Essex (County of Essex, 2020) and imagery collected and shared by Point Pelee National Park.

We used our final house counts (i.e., our estimated maximum counts) at both Pelee and Matchedash in all survey years to compute an average house count at each site for the contemporary survey period. Because we chose to use our estimated maximum house counts that were based on a combination of aerial and ground census methods, rather than our minimum ground‐only observations (which were incomplete in some cases), we are confident that we have not underestimated the current muskrat population at either site by potential detection errors (i.e., missed houses) resulting from site conditions or incomplete survey coverage.

Historic house counts dating back to 1957 are available for Pelee; however, a detailed breakdown of the count results by survey zone was not provided until 1967. Examining these data, we realized that not all areas of the marsh were surveyed consistently from year to year and that some years suffered from poor survey conditions (e.g., due to deep snow), which likely decreased house detection rates. Because we could not supplement the historic field data with imagery as we did for our recent surveys, we decided that a trend over time analysis of house counts would be more accurate if we omitted the survey years and zones where survey coverage was reportedly poor, incomplete, or simply unknown due to a lack of information in historic reports. We therefore began our analysis of the Pelee muskrat population with the 1968 data and removed the years 1977–1978, as well the survey zones 14 and 15 in all years, from our final analyses to create a reduced but more consistent and comparable set of time‐series data. However, we also computed a mean house count for the 1968–1980 period at Pelee without excluding the two survey zones to compare with our final results. At Matchedash, survey coverage was generally more consistent from year to year (except for 1981, which was surveyed by boat in early spring, and 1985 when poor ice conditions limited travel over some areas of the marsh), and thus, we used all seven survey years and the annual results obtained for the entire survey area to calculate a mean house count for the historic period at that site.

As was done for the historic surveys, we used our annual counts of muskrat houses as indices of abundance for each site, a common method first described by Dozier (1948) and since undertaken by many researchers as a means to infer abundance and track annual change in muskrat populations (e.g., Greenhorn et al., 2017; Kroll & Meeks, 1985; Proulx & Gilbert, 1984; Toner et al., 2010).

## 3 RESULTS

### 3.1 Point Pelee Marsh

**FIGURE 4:**
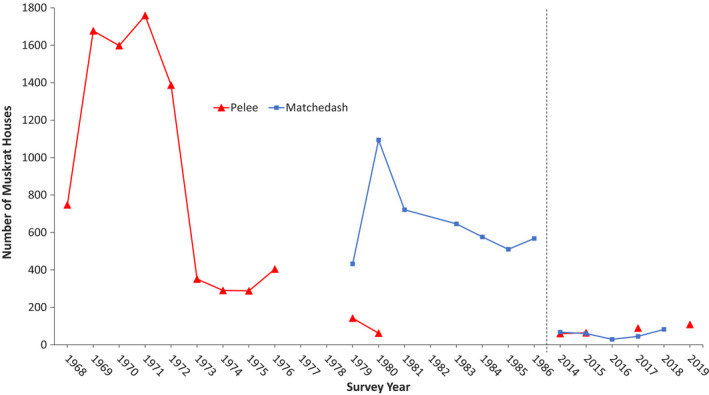
Historic and contemporary house counts for Point Pelee (all survey zones, but complete survey years only) and Matchedash Bay–Gray Marsh using replicated survey methods

We found very low numbers of muskrat houses at Pelee over our four contemporary survey years between 2014 and 2019 as compared to the historic survey period from 1968 to 1980 (Figure 4). The mean (SE) number of muskrat houses found at Pelee between 1968 and 1980, excluding incomplete survey zones and years, was 719 (202.9). The 95% confidence interval for house counts during these years was 267–1171. In contrast, we derived a mean (SE) house count of 47 (7.4) from our 2014–2019 surveys, excluding the same zones as we did with the historic data set. The 95% confidence interval for the house counts during these latter years was 24–71.

**FIGURE 5:**
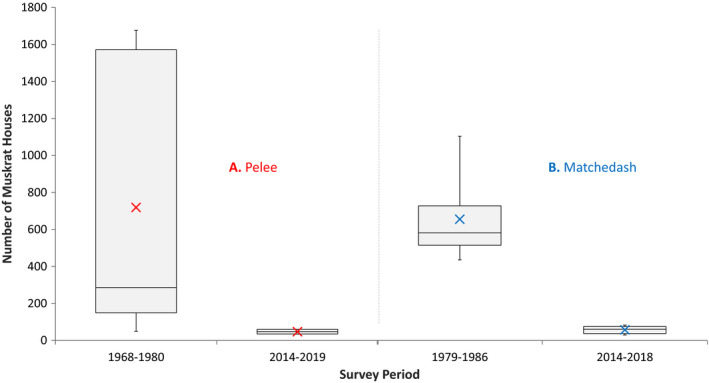
Box plots of annual muskrat house counts at **A**. Point Pelee (consistently surveyed zones and years only) and **B**. Matchedash Bay for each survey period. The horizontal line inside each box represents the median house count, and the mean count for each is denoted by an X. Lower and upper box boundaries represent the 25th and 75th percentiles of the data, respectively. Whiskers denote the minimum and maximum house counts. Estimated maximum counts were used to compute the results for the contemporary survey period at each site

Our contemporary surveys of Pelee demonstrated a 93% decline in the mean number of muskrat houses from historic levels 40–50 yrs prior (Figure 5). Retaining the count results for the excluded survey zones still resulted in a dramatic difference in the mean number of houses found between each survey period (791 historically vs. 80 recently) and a 90% decline in house numbers. Excluding the imagery‐based estimates that we added for the areas we could not access, our mean (SE) house count from the ground count data alone was 50 (6.9) for all survey zones and 39 (6.5) for the standardized zones, representing declines of 94% and 95%, respectively, from historic times.

### 3.2 Matchedash Bay–Gray Marsh

Similar to Pelee, we found relatively low numbers of muskrat houses at Matchedash in each of our five recent survey years from 2014 to 2018 when compared to the historic survey period from 1979 to 1986 (Figure 4). The mean (SE) number of muskrat houses found at Matchedash between 1979 and 1986 was 650 (81.9). The 95% confidence interval for house counts averaged across these years was 450–850. In contrast, we observed a mean (SE) house count of 57 (9.1) during 2014–2018, using our estimated maximum house counts derived from a combination of ground and imagery‐based surveys. The 95% confidence interval for the house counts averaged across these latter years was 32–82.

Our contemporary surveys of Matchedash demonstrated a 91% decline in the mean number of muskrat houses from historic levels 30–40 yrs prior (Figure 5). Excluding the imagery‐based estimates that we added for the areas we could not access, our strictly ground‐based survey results showed an even greater decline of almost 95%, as our mean (SE) house count from those data alone was 35 (5.8).

## 4 DISCUSSION

Our surveys demonstrate substantial declines in muskrat populations at two large coastal wetlands in Ontario based on direct field observations spanning 40–50 yrs. The data we present are not confounded by trapper effort like harvest data often are, but they correspond to reported trends in provincial harvest data and are consistent with anecdotal evidence of muskrat decline. Ahlers and Heske (2017) called for direct evidence of muskrat population trends to avoid confounds with harvest effort, and we provide such evidence here.

It has been suggested that most survey techniques for muskrats are only feasible across small geographic extents and may not be useful for examining large‐scale population trends (Roberts & Crimmins, 2010). While this may be true, we have two independent data sets from widely separated wetlands illustrating declines of similar magnitude over a similar time frame, which are also coincident with declines in our provincial harvest data over the same approximate time period. Although our findings are limited to two individual wetlands and therefore extrapolating the trends to a broader region would be premature, when considered alongside the provincial harvest records our data are consistent with a broader trend of muskrat decline across the Great Lakes basin in Ontario. It would be worthwhile to discover and collect additional survey data from other areas to shed more light on the scale of the observed trends.

The greater than 90% decline in muskrat house abundance at each of our study sites is startling, yet we are confident that the survey data indicate a true decline of this magnitude. In many wildlife surveys, there is potential for overestimating or underestimating abundance as a result of observer error. In the case of muskrat house surveys, some houses may be missed, and some structures may be falsely labeled as active houses when they are in fact only feeding structures or inactive houses, or vice versa. However, we assume that in our study, on average, detection probabilities were similar from year to year (except for the survey years that were deliberately eliminated from the analysis because the surveyors reported poor conditions that hindered their ability to detect houses) and that in most cases muskrat structures were correctly identified. We attempted to replicate the historic survey methods as closely as possible so that the results we obtained could be largely attributed to actual population change and not simply methodology differences. Furthermore, considering that our final house counts for the recent survey years at each site were adjusted based on imagery data that were used to supplement our ground counts, and it is more difficult to distinguish new houses from old ones on aerial photographs, it is more likely that we have overestimated contemporary muskrat abundance by including houses in our tally that are no longer in use. Therefore, our estimates of the magnitude of muskrat population change at each site are likely conservative.

Muskrat numbers have been shown to fluctuate periodically; thus, it is possible that low house counts may be reflecting a low phase in a normal population cycle. Erb et al. (2000) examined ninety‐one historic time series of muskrat harvest data from boreal, taiga, and southern Arctic regions of Canada and found overall that mean period length ranged from 3.7 to 8.6 yrs. If the same pattern holds true in southern Ontario and the start of our surveys corresponded to a population low phase, we would expect to have seen a greater increase in our recent house count numbers over the 5–6 yrs we conducted the surveys. Based on a visual examination of the data, we observed no such increasing trend, and similarly, there is no increasing trend evident in muskrat harvest data collected over the same time period. Instead, it appears that muskrat populations at Point Pelee and Matchedash Bay–Gray Marsh are currently persisting at very low levels and are much reduced from the levels seen 30–50 yrs ago. Furthermore, harvest data suggest that these declines are widespread.

### 4.1 Reasons for decline

It is unclear whether the apparent declines in muskrat abundance that we have demonstrated are a result of a broad‐scale underlying cause or site‐specific factors.

In recent studies of muskrat occurrences in Lake Ontario–St. Lawrence River coastal wetlands, it was found that water‐level regulation of that system had a negative influence on muskrat abundance (Greenhorn et al., 2017; Toner et al., 2010). While the two Great Lakes that our study sites occur on are not subject to such tightly controlled water levels as Lake Ontario, both Lake Erie and Lake Huron have experienced record high and extended low water levels over the duration that our investigation spans (NOAA, 2020), primarily brought on by weather extremes and climate variability over the past few decades (Gronewold & Rood, 2019). The presence of water, either through excessively high water levels or periods of drought, has been considered the greatest selection pressure affecting muskrats (Ahlers et al., 2015; Bellrose & Brown, 1941; Errington, 1963; Proulx & Gilbert, 1983; Virgl & Messier, 1996; Ward & Gorelick, 2018). Essentially, the water level in a marsh is suitable for muskrats when it is deep enough to maintain travel routes and allow access to houses and feeding areas underwater (even in cold winters when ice cover can be very thick), yet shallow enough to permit the growth of emergent aquatic vegetation. It is also important for muskrats that any changes in water level occur gradually, so that houses are neither flooded out nor left high and dry. We have not conducted a detailed analysis of water‐level patterns at Pelee and Matchedash in relation to muskrat occupancy; therefore, this is an avenue that warrants closer examination.

Closely related to water levels is the hypothesis that habitat change has led to dramatic reductions in muskrat numbers. For example, Ward and Gorelick (2018) suggested that the loss of critical wetland habitat due to drying is the primary driver responsible for the decline of muskrats in the Peace–Athabasca Delta over the past half‐century and potentially across their entire native range. While the amount of wetland habitat on the landscape available to muskrats is certainly an important factor influencing their population levels, the sizes of the wetlands in our study area have not changed appreciably over the time span of our investigation. Province‐wide, Ontario has experienced significant loss of wetlands over the past two centuries, but most of this loss (70% of original wetland area) occurred between the time of European settlement (circa late 1700s) and 1982 (Ontario Biodiversity Council, 2010). Only a relatively small additional amount of wetland area loss (3.8%) occurred between 1982 and 2014 (Ontario Biodiversity Council, 2015). Thus, it is clear that the vast majority of wetland loss in Ontario occurred prior to the recent period of muskrat decline that we have demonstrated. Therefore, it seems unlikely that a loss of habitat area is a major factor driving the recent declines we have seen in muskrat populations in Ontario over the past 30–50 yrs.

A more plausible explanation for muskrat decline in Ontario is a change in the overall quality of muskrat habitat, particularly in the structure and composition of Great Lakes coastal wetlands that were previously occupied in large numbers by muskrats as shown in this study. Proulx and Gilbert (1983) demonstrated that muskrats prefer to occupy areas of marsh with a 1:1 interspersion of emergent vegetation and open water, a structural pattern of habitat patchiness often referred to as a “hemi‐marsh.” Yet, there is evidence around the Great Lakes basin that the structural diversity of wetlands has declined. The research of Wilcox et al. (2008) has shown that Lake Ontario coastal wetlands have experienced a significant decrease in both emergent marsh habitat heterogeneity and the amount of emergent‐open water edge in the past 50 yrs since regional water‐level control was implemented. At the Point Pelee Marsh, one of our two study sites, Markle et al. (2018) demonstrated that both marsh habitat diversity and open water connectivity have declined since 1931, with the most significant reductions occurring between 1959 and 2015. Similarly, at our other study site, the Matchedash Bay–Gray Marsh, aerial photograph interpretations by Taylor et al. (2015) demonstrate a large increase in the areal coverage of dense emergent vegetation along with a correspondingly large decrease in the extent of aquatic habitat between the years of 1973 and 2008. These reductions in habitat structural quality coincide with the time periods over which we have demonstrated a decline in muskrat abundance at both of our study sites.

The research of Markle et al. (2018), Wilcox et al. (2008), and others (e.g., Farrell et al., 2010; Wilcox et al., 2003) also demonstrates changes in the compositional quality of Great Lakes coastal wetlands over the past 50 yrs that coincide with the decline in muskrats that we report here. Not only have they demonstrated class‐level habitat changes, such as the expansion of dense emergent cattail marshes at the expense of meadow marsh and other shallow mixed marsh communities, but also they have documented dramatic increases in the prevalence of invasive emergent marsh species such as *Phragmites australis* subsp. *australis, Typha angustifolia,* and *Typha* X *glauca*. In particular, the hybrid species of cattail (*Typha* X *glauca)* has increased significantly in extent, displacing both the native broad‐leaved cattail (*Typha latifolia*) and other aquatic plant species, and is now the dominant emergent species in most of our coastal wetlands.

The well‐documented changes in both the structure and composition of coastal wetlands over the past 50 yrs may have contributed to the dramatic decline in muskrats at our study sites, yet this remains unclear. In the absence of an experimental study or control sites where such habitat changes have not occurred and muskrat numbers have not declined, it would be premature to suggest that this type of habitat change is the underlying mechanism that has driven muskrat declines across a broader regional scale. Therefore, this hypothesis warrants further investigation in future studies looking at changes in muskrat abundance.

Several other explanations for the decline in muskrat populations are possible but seem less likely for a variety of reasons. Overharvest by trappers is often postulated as the reason behind furbearer population declines; however, the demographic characteristics of muskrats make them resilient to overharvest (Boutin & Birkenholz, 1987). Even when heavy harvest does occur in a wetland, the muskrat's high reproductive output and dispersal ability usually enable a population to rebound relatively fast. As well, one of our study sites (Pelee) has been closed to muskrat trapping since 1959; therefore, muskrat harvest levels have had no direct influence on the population dynamics there for at least 50 yrs. At our other study site, local long‐time trappers have reported low harvests and great difficulty finding muskrats for many years now (M. Dunlop, personal communication, March 2014).

Predation and disease are important natural aspects of muskrat ecology that can affect their abundance over the short term, but which typically balance out over the long term. Errington (1963) suggested that significant mortality of muskrats due to predation usually only occurs when muskrats are already vulnerable as a result of disease or habitat changes such as loss of cover, drought/flooding, or freeze‐outs. As well, muskrat populations typically respond to predation mortality by a variety of compensatory processes, such as increased reproduction and survival among the remaining individuals (Errington, 1951). Mink (*Neovison vison*) are considered the most important predator of muskrats (McDonnell, 1983), yet there is evidence from harvest data of long‐term mink population decline in Ontario (Gorman, 2007) and no evidence of local mink population booms at our study sites (personal observations). Disease outbreaks among muskrats are typically localized and usually occur when muskrat populations are under some form of stress, such as overcrowding, food shortage, or drought (Errington, 1963). Also, as with predation, the effects of disease on muskrat population size are often short‐term because of compensatory responses in other sources of mortality or in reproduction. We are unaware of any documented outbreaks of disease in muskrats at either of our study sites or elsewhere in Ontario over the time period that our study covers (Ganoe et al., 2020).

### 4.2 Implications of muskrat decline

A marked decline in muskrat populations is concerning not only for the value of muskrats in their own right, but also for the broader economic, ecological, and cultural benefits that muskrats and wetlands provide. Muskrats are highly valued by trappers and are an important part of our natural heritage. In many Indigenous cultures, the muskrat is revered for the life‐giving role it is said to have played in the creation of the Earth as we know it. As such, a loss of muskrats comes with significant costs, many of which we might already be experiencing (Papworth et al., 2008).

Muskrats manipulate marshes (Higgins & Mitsch, 2001), promoting greater diversity and providing ecological services to humans and other wetland species. Many of the species that benefit from muskrats are of conservation concern themselves. Thus, the loss of muskrats from our marshes may compound existing problems within these wetlands and across broader landscapes containing wetlands. Understanding the current state of the muskrat might help us to better understand the current state of our wetland ecosystems.

There is a clear need for more research on the relationship between muskrat populations and the various factors that influence their abundance, especially if we are to understand what has led to their recent declines and if we wish to prevent further loss of this iconic species.

## CONFLICT OF INTEREST

The authors declare there are no competing interests or conflicts of interest.

## AUTHOR CONTRIBUTIONS

**Carrie Sadowski:** Conceptualization (equal); data curation (lead); formal analysis (lead); funding acquisition (supporting); investigation (lead); methodology (lead); project administration (lead); resources (lead); supervision (equal); validation (equal); visualization (equal); writing—original draft (lead); writing, reviewing, and editing (equal). **Jeff Bowman:** Conceptualization (equal); data curation (supporting); formal analysis (supporting); funding acquisition (lead); investigation (supporting); methodology (supporting); project administration (supporting); resources (supporting); supervision (equal); validation (equal); visualization (equal); writing—original draft (supporting); writing, reviewing, and editing (equal).

## DATA AVAILABILITY STATEMENT

Data are archived in the Dryad Data Repository at https://doi.org/10.5061/dryad.b2rbnzsf2.
